# Neutrality in plant–herbivore interactions

**DOI:** 10.1098/rspb.2023.2687

**Published:** 2024-02-21

**Authors:** Vincent S. Pan, William C. Wetzel

**Affiliations:** ^1^ Department of Integrative Biology, Michigan State University, Easting Lansing, MI 48824, USA; ^2^ Ecology, Evolution, and Behavior Program, Michigan State University, Easting Lansing, MI 48824, USA; ^3^ W. K. Kellogg Biological Station, Michigan State University, Hickory Corners, MI 49060, USA; ^4^ Land Resources and Environmental Sciences, Montana State University, Bozeman, MT 59717, USA

**Keywords:** herbivory, neutral theory, functional equivalence, intraspecific variation, sub-individual variation, R package

## Abstract

Understanding the distribution of herbivore damage among leaves and individual plants is a central goal of plant–herbivore biology. Commonly observed unequal patterns of herbivore damage have conventionally been attributed to the heterogeneity in plant quality or herbivore behaviour or distribution. Meanwhile, the potential role of stochastic processes in structuring plant–herbivore interactions has been overlooked. Here, we show that based on simple first principle expectations from metabolic theory, random sampling of different sizes of herbivores from a regional pool is sufficient to explain patterns of variation in herbivore damage. This is despite making the neutral assumption that herbivory is caused by randomly feeding herbivores on identical and passive plants. We then compared its predictions against 765 datasets of herbivory on 496 species across 116° of latitude from the Herbivory Variability Network. Using only one free parameter, the estimated attack rate, our neutral model approximates the observed frequency distribution of herbivore damage among plants and especially among leaves very well. Our results suggest that neutral stochastic processes play a large and underappreciated role in natural variation in herbivory and may explain the low predictability of herbivory patterns. We argue that such prominence warrants its consideration as a powerful force in plant–herbivore interactions.

## 1. Introduction

**Figure 1.**
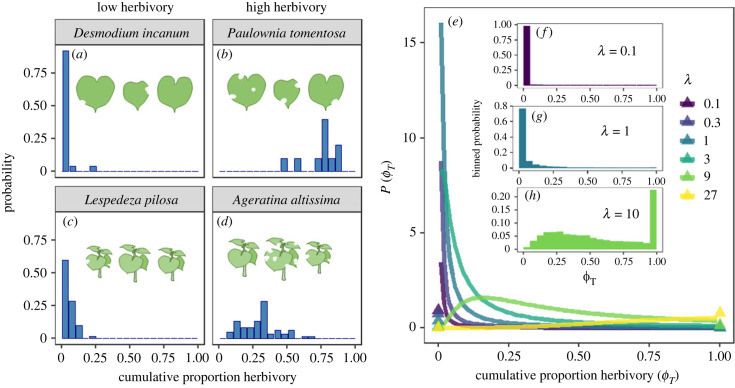
Example empirical distributions of among-leaf (*a,b*) and among-plant (*c,d*) herbivore damage in the HerbVar dataset. (*e*) The probability density function of cumulative proportion herbivore damage *ϕ_T_* according to the neutral model with different attack rates *λ*. The two probability point masses at 0 and 1 are indicated by triangles. To better visualize the mix of discrete and continuous probabilities, we plotted the binned probability of different herbivore damage classes at 5% intervals for different values of attack rates (*f–h*).

The distribution of herbivore damage has long been noted for its tremendous variability across time and space, and among leaves and individual plants [1–3] (figure 1*a*,*d*). This variability has crucial ecological and evolutionary consequences, including reducing plant tolerance of herbivory [4], strengthening herbivore interactions [5], and modifying herbivore community assembly [6]. Importantly, it is a prerequisite for natural selection that gave rise to the plethora of plant defence phenotypes in nature. Explanations for such heterogeneity have conventionally been attributed to heterogeneity in plant quality [7–9] or interactions among herbivores [10,11], and most of the literature on plant–herbivore biology indeed seeks deterministic, trait-based predictors of herbivore damage patterns within and across plants. Despite great advances in our understanding of plant–herbivore interactions [12], however, the explanatory power of well-established plant traits remains surprisingly and consistently low [13].

In face of the challenge of predicting herbivore damage patterns, the field has generally proceeded as if more research will reveal the key trait-based processes governing herbivore damage patterns. An alternative hypothesis is that predicting herbivory has been difficult because the process of herbivory contains much inherent neutral stochasticity that is unrelated to heterogeneity in plant quality and interactions among herbivores [14,15]. Recent work has highlighted the importance and dominance of process-based stochasticity in lifetime reproductive success [16,17], but we still have a poor understanding of its role in plant–herbivore ecology and evolution. For instance, patterns of herbivore damage are often used as evidence for an array of biological phenomena, including induced plant defences [2,18], informed herbivore movement and plant communication [19], variation in plant defence [1] and selection regimes [20], without testing if those patterns could have arisen from process stochasticity. A key advance that would improve our ability to distinguish between herbivore damage patterns caused by plant traits and purely neutral stochastic processes is a process-based null model for damage distributions based on first principles. Such a model could characterize damage distributions under functional neutrality, allowing for the evaluation of mechanisms omitted from the model and generate testable predictions for a wide range of behaviours, not previously recognized as being related.

Here, we derive a neutral process-based model of herbivore damage using simple and realistic assumptions about the accumulation of damage on leaves from randomly sampled herbivores from a regional species pool. Our model accords with the recent push to integrate stochasticity in community ecology [21] and represents a mere logical extension to well-established macroecological laws on metabolic allometry [22–25]. We then compare our model predictions to global observations of herbivore damage patterns for 496 species using data from the Herbivory Variability Network. As a foil to common wisdom, our model assumes that plants are identical, unchanging, passive and fed upon randomly by herbivores. This means plants do not exhibit induced defences or differ in palatability, and herbivores do not aggregate or repel each other. We focused on the distribution of damage at the among-leaf (leaves belonging to the same individual) and among-plant scales (conspecific individuals in a population), with emphasis on the level of variability as quantified by the summary statistic, coefficient of variation (CV). We chose this feature of the distribution to focus our discussion on because recent work highlighted variation in plant–herbivore interactions in eco-evolutionary outcomes [26]. However, we seek to predict and describe the entire distribution. Our goal is not to test whether plant–herbivore interactions are neutral⁠—they are not—but to identify the manners and conditions where our *a priori* neutral expectations succeed or fail to match reality [27]. We addressed three main questions: (1) Can a neutral model generate the high variation in herbivore damage common in nature [3,26] and predict changes in this variation? (2) How does the neutral model deviate from observed damage patterns? (3) What explains model deviations? We might expect model deviation to be predicted by plant traits more than environmental variables for instance, if plants evolved traits that altered the neutral dynamics of herbivore damage. Taken together, our study seeks to identify key ecological processes in the generation of herbivore damage that give rise to emergent patterns shaping plant–herbivore interactions.

## 2. Results

### (a) Neutral model

First, we derive a probability distribution of herbivore damage at the among-leaf and among-plant scales. Our model assumes that the observed proportion damage on a leaf or plant represents the accumulation of leaf area removed over a number of discrete feeding events. At each feeding event, the proportion leaf area removed increases with the whole-body metabolic rate of a randomly sampled herbivore individual from a regional herbivore pool. This metabolic rate increases with the body size of the herbivore. In our model, larger herbivores are less abundant and less speciose within this regional pool.

Thus, the cumulative proportion damage *ϕ_T_* (hereafter as ‘proportion damage') is the sum of proportion leaf area loss in all events *k* until the whole leaf is consumed. Adhering to our assumption of neutrality, we assume the number of feeding events *k* follows a Poisson distribution with an identical rate *λ* across all leaves and the occurrence of each feeding event is independent. The distribution of cumulative proportion damage *ϕ_T_* is therefore a truncated compound Poisson distribution:

2.1 $$\phi_{T}(k)=\{\begin{array}{ccc} \overset{k}{\underset{i}{\sum }}\phi_{i}, & \;\overset{k}{\underset{i}{\sum }}\phi_{i} & \leq 1\\ 1,\;\; & \overset{k}{\underset{i}{\sum }}\phi_{i} & >1. \end{array}$$

Let *ϕ* be the proportion leaf area removed by a randomly sampled herbivore from a regional herbivore pool in a single feeding event, with a lower and upper bound *ϕ_m_* and *ϕ_M_*, respectively. Macroecological studies have shown that many important biological variables, including population density [22], species richness [23,24] and metabolic rate [25], scale as a power law with body size with specific allometric coefficients. Assuming the energy requirement of a herbivore is proportional to leaf area consumption, a probability density function of *ϕ* with allometric coefficient *α* = 14/9 can be derived from these scaling laws (see electronic supplementary material, appendix, model derivation and numerical approximation):

2.2 $$P(\phi )=\frac{1-\alpha }{\phi^{\alpha }(\phi_{M}^{1-\alpha }-\phi_{m}^{1-\alpha })}.$$

Equation (2.2) is also known as the truncated Pareto distribution and models the probability of drawing some proportion leaf area removed in a feeding event. The bounds of *ϕ* can be chosen *a priori* and are discussed further in §4a*.* Hence, the distribution of cumulative proportion damage can be fully described by a single unknown parameter, the attack rate by herbivores *λ*. In electronic supplementary material, appendix, model derivation and numerical approximation, we extend this model to the presence/absence of damage, which unlike (2.1) can be expressed in closed form.

We found that stochastic sampling of different sizes of herbivores from a regional pool alone is sufficient to generate realistic herbivore damage distributions with high degrees of variability (figure 1*e–h*). This variability also changes predictably along herbivory intensity. For analytical tractability, we assume proportion damage *ϕ_T_* ≪ 1 (e.g. a plant with many leaves), though the results are qualitatively similar in simulations that relax this assumption (electronic supplementary material, appendix, asymptotic behaviour). As a repeated additive process, the central limit theorem ensures that proportion damage becomes approximately normally distributed when the attack rate *λ* becomes very large [28]. Therefore, given a large *λ* and a generic unitless inequality index I, such as the Gini index ($c=\sqrt{\pi }$), CV (*c* = 1), or Hoover index ($c=\sqrt{2\pi }$), the following holds (electronic supplementary material, appendix, asymptotic behaviour):

2.3 $$I[\phi_{T}]\approx \frac{\sqrt{E[\phi^{2}]}}{c\sqrt{\lambda }E[\phi ]}.$$

Hence, any variable associated with a greater attack rate, or similarly, mean herbivore damage, should also be associated with a lower measure of variability, declining by a factor $1/\sqrt{\lambda }$, all else being equal. Examples of reported associations between damage variability and predictors of interest (e.g. time of year, plant size, herbivore density) may therefore as well be explained by their associations with mean damage (electronic supplementary material, table S1). We thus caution the interpretation of damage variability without considering the mean–variance relationship and ‘statistical pleiotropy' more broadly, where one factor affects multiple features of a statistical distribution.

### (b) Comparing neutral and observed distributions

Next, we compared the predictions of the neutral model to a global empirical dataset of proportion herbivore damage at the among-leaf and among-plant scales. The dataset was contributed by greater than 160 researchers from the Herbivory Variability Network and consisted of 765 field surveys of 496 species, spanning across 116° of latitude and all nine Whittaker biomes (electronic supplementary material, appendix, HerbVar dataset, figure S1). All surveys were collected using the same protocol, where a target of 30 randomly selected conspecific plant individuals and their nearest conspecific neighbour within a population were visually assessed for height and proportion damage on ten randomly selected leaves. Plant level damage was estimated as the average damage across all sampled leaves belonging to the plant. We only included surveys with more than 15 records for among-plant damage, and plants with more than 10 records for among-leaf damage. For each set of herbivory observations at the among-leaf or among-plant scale, we parametrized our model by estimating a single unknown parameter, the attack rate *λ*, from the data.

**Figure 2.**
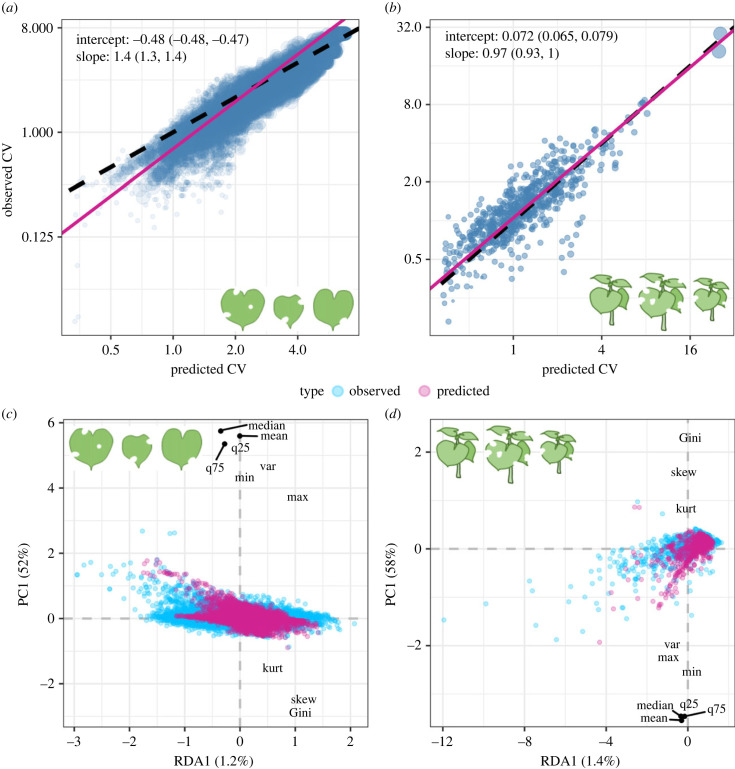
Observed versus predicted coefficient of variation of among-leaf (*a*) and among-plant (*b*) herbivore damage. Slope and intercept are taken from a major axis regression model and plotted as a red line. The 95% confidence intervals are displayed in parentheses. Each point represents a distribution, with the size scaled to the sample size of the distribution. Points above the one-to-one black dashed line indicate overprediction of CV. Similarly, points below the dashed line indicate underprediction of CV. The first constrained and unconstrained axes of RDA of among-leaf (*c*) and among-plant (*d*) statistical probes. Each point represents a distribution. High similarity in the shape of the predicted and observed distributions is demonstrated by the high overlap in the predicted and observed data clouds.

We found that the neutral model with only one degree of freedom, the herbivore attack rate *λ*, can generate herbivore damage distributions that are very similar to observed damage patterns (table 1 and figure 2). Among-leaf damage, however, resembled neutral patterns more closely than among-plant damage. We examined how closely the neutral model approximates observed data across three absolute measures of model fit. Our neutral model generated CVs that closely matched observed among-leaf CVs (*r*^2^ = 0.86) and somewhat less closely matched among-plant CVs (*r*^2^ = 0.70). A very low proportion of variance in ten statistical probes (summary statistics of a distribution *sensu* [29]) is attributed to whether a distribution is predicted or observed (*R*^2^ = 0.012) among leaves, but this was slightly higher among plants (*R*^2^ = 0.014). Likewise, a low proportion of Kolmogorov–Smirnov (KS) tests found significant differences between observed and neutral distributions among leaves (1.6%), but this was higher among plants (22%). Next, we examined the relative fit of the neutral model against alternative phenomenological non-neutral hypotheses, allowing for strong inference (i.e. model selection) [30]. We note that these alternative hypotheses are less parsimonious, having three or four times as many degrees of freedom as our simpler neutral model. They also provide only phenomenological, not mechanistic or dynamical, insight. That is, they use patterns, as opposed to underlying mechanisms, as model parameters; hence, unlike our neutral model, they provide no testable auxiliary predictions beyond the patterns of herbivore damage. Nonetheless, they represent commonly used alternatives against which to compare our first principles neutral model. At the among-leaf scale, the neutral model found significantly more support than a hurdle truncated lognormal (HTLN) (median *Δ*AIC_C_ = 2.3) and zero-one-inflated beta model (ZOIB) (median *Δ*AIC_C_ = 4.5), or non-process-based null models, for most of the observed damage distributions (table 1). By contrast, at the among-plant scale, the neutral model generally found less support than alternative null models (HTLN median *Δ*AIC_C_ = −16; ZOIB median *Δ*AIC_C_ = −9.5). These results suggest that our neutral model adequately describes most natural damage patterns and may therefore serve as a useful reference with which we compare observed data against. We proceed to investigate how neutral patterns deviate from empirical patterns below.

**Table 1.** Median and 95% quantiles of measures of model fit. CV *r*^2^ corresponds to the proportion variance in observed log CV explained by predicted log CV. Constrained *R^2^* corresponds to the proportion of variance in ten statistical probes explained by whether the distribution is predicted or observed in an RDA. At the among-leaf scale, this is the proportion of remaining variance after removing 41% of total variance attributed to individual survey identity. *Δ*AIC_C_ show the relative fit of the neutral model compared to generic non-neutral null models of herbivore damage, a three-parameter hurdle truncated lognormal (HTLN) and four-parameter zero-one-inflated beta (ZOIB) distribution. Lower values indicate better fits for all metrics except CV *r*^2^ and *Δ*AIC_C_, for which higher values indicate better fits.

	**among leaves**	among plants
KS statistic	0.20 [0.2, 0.50]	0.18 [0.067, 0.43]
% KS test *p* < 0.05	1.6 [1.5, 1.8]	22 [20, 24]
CV *r^2^*	0.86	0.70
constrained *R^2^*	0.012	0.014
*Δ*AIC_C_ (HTLN: neutral)	2.3 [−19, 12]	−16 [−290, 14]
*Δ*AIC_C_ (ZOIB: neutral)	4.5 [−32, 17]	−9.5 [−280, 21]
distribution sample size	13 [10, 51]	60 [28, 82]
total distributions	23 648	739

Leaves within a plant sustained more similar damage levels to each other and individual plants within a population sustained damage levels more different from each other than expected by the neutral model. We evaluated this result by comparing the CVs of observed damage distributions against distributions generated by our neutral model and by a randomization procedure. Because leaves with similar levels of damage may be more likely to belong to the same plant individual and each plant individual has slightly different expected damage, we can break the non-random associations by shuffling observed leaves within a survey among plants, thereby generating an empirical null distribution (hereafter as ‘shuffled' distribution). A null distribution generated this way avoids making assumptions about the data generating process and serves a second line of evidence alongside our neutral model. At the among-leaf scale, observed CV is 5.1% lower than the shuffled CV (*Z* = −29, *p* < 0.0001) and 15% lower than the neutral model predicts (*Z* = −87, *p* < 0.0001), and the deviation is greatest at lower CV values (figure 2*a*). Observed damage values tend to be less variable, less skewed, less leptokurtic, and less extreme than the neutral model expects (electronic supplementary material, table S2; figure 2*c*). By contrast, at the among-plant scale, observed CV is 26% higher than the shuffled CV (*Z* = 18, *p* < 0.0001) and 7.0% higher than the neutral model predicts (*Z* = 5.3, *p* < 0.0001; figure 2*b*). Observed damage values tend to be more variable, more skewed, more leptokurtic, and more extreme than the neutral model expects (electronic supplementary material, table S2; figure 2*d*). These results further corroborate that among-leaf damage is more even than expected by neutral dynamics and that damage is more aggregated among plants (electronic supplementary material, figure S2).

### (c) Predictors of neutral deviations from observations

Finally, we found the deviations of neutral patterns from observed patterns varied with plant size and phylogeny, but not with geography and climate. We measured the deviation of neutral patterns from observations in terms of Kullback–Leibler (KL) divergence, a measure of relative entropy, which can be interpreted as the expected additional amount of information needed to have the neutral model fully agree with observed data (i.e. knowledge gap). We detected a moderate phylogenetic signal in KL divergence at both scales (among-leaf: Pagel's *λ* = 0.38; among-plant: Pagel's *λ* = 0.42; table 2; electronic supplementary material, figure S3), suggesting unmeasured plant traits may underlie different non-neutral damage patterns. Indeed, larger plants had greater deviations at the among-leaf scale, albeit with little explanatory power (table 2). By contrast, KL divergence is not predicted by latitude, mean annual temperature and mean annual precipitation (table 2).

**Table 2.** Mean posterior standardized marginal effects (nat/s.d.) and *ΔR*^2^ of KL divergence of neutrality from observation. The 95% credible intervals are shown in brackets (in bold if the intervals do not overlap zero). Species phylogeny accounts for phylogenetic relatedness, whereas species ID accounts for species specific environmental or niche effects.

	among leaves		among plants	
	standardized *β*	*ΔR*^2^	standardized *β*	*ΔR*^2^
PI	—	0.28 [0.13, 0.63]	—	0.19 [0.065, 0.43]
survey ID	—	0.27 [0.12, 0.63]	—	—
species ID	—	0.19 [0.057, 0.54]	—	0.23 [0.061, 0.56]
phylogeny	—	0.14 [0.036, 0.47]	—	0.083 [0.0038, 0.30]
MAT	0.11 [−0.067, 0.28]	0.011 [1.6 × 10^−6^, 0.042]	0.054 [−0.076, 0.18]	0.0074 [0, 0.031]
MAP	0.021 [−0.063, 0.11]	0.0031 [0, 0.014]	−0.035 [−0.10, 0.030]	0 [0, 0.0050]
| latitude |	0.069 [−0.086, 0.23]	0.0084 [0, 0.039]	0.021 [−0.10, 0.22]	0.0021 [0, 0.016]
log plant height	**0.019 [5.8 × 10^−5^, 0.039]**	0.0011 [2.6 × 10^−6^, 0.0026]	0.030 [−0.040, 0.099]	0.0010 [0, 0.0066]
residual	—	0.26 [0.25, 0.26]	—	0.35 [0.31, 0.40]
Pagel's *λ*	0.38 (*p* = 0.04)		0.42 (*p* < 0.001)	

## 3. Discussion

We show that the distribution of herbivore damage, owing to the simple process of sampling herbivores with different body sizes from the regional herbivore pool, can be highly unequal among leaves and among plants. This is despite the neutral assumption that plants are identical, unchanging, passive, and fed upon randomly by herbivores. Stated another way, while observed patterns of herbivore damage are the amalgamation of herbivore pressure and plant defences that might vary across individual leaves and plants and are important in many systems, neither process needs to be invoked to explain the distribution of damage across individual leaves and individual plants. Rather, the distribution of damage, being an emergent pattern of many interacting plants and herbivores, is largely governed by a set of simple underlying stochastic processes, regardless of the details of the system.

We have identified this minimum process by which herbivory occurs, and on top of which the complexity of traits and herbivore behaviour then play out. Although plant traits may account for where the neutral model deviates from observed damage patterns, the extent of the deviations is surprisingly limited. Indeed, processed-based stochasticity is prevalent in nature [31] and may play a large role in plant–herbivore interactions (table 2). Yet, the role of stochasticity has been largely ignored in the plant–herbivore literature that emphasizes traits and evolution. Our study highlights how such a perspective shift may provide unique insights that apply generally to plant–herbivore interactions across broad taxonomic and geographical coverage.

That there is inherently high heterogeneity in herbivore damage has two major implications. First, it implies that herbivory pressure on plants has low predictability and can lead to the common observation that plant defence allocation fails to match the cost of herbivory [32]. Information from ongoing herbivore damage is thus crucial to deal with future attacks, as exemplified by the prevalence of short-range inter-plant communication [33], localized induced defences, and phenotypic plasticity [34,35]. Second, it challenges the common assumption that variation in damage contains much biological information of unmeasured drivers. Our model suggests that whether a plant individual within a population sustains high or low damage is mostly driven by luck, or it is at least indistinguishable from luck, and the plant has limited ability to affect its herbivore damage. Taking this view, we may recognize the importance of herbivory, where diverse plant defence traits nevertheless evolved [36], despite the insensitivity of herbivore damage to trait differences and the dominance of genetic drift that reduces selection efficiency [16]. We may also recognize that trait-based approaches to predicting plant herbivory can only take us so far, and to its limited extent, existing studies have succeeded spectacularly in characterizing most non-neutral variation in damage that can be explained. Low *R*^2^ values in herbivory studies [13] are to be expected when damage contains high neutral process variance. While even very small differences in damage or fitness outcomes due to some traits represent strong selection differentials that may lead to rapid evolution of said traits, this does not imply the traits are predictive of damage patterns, and raises the question whether trait-based models are necessary to understand variations in damage, or interactions and fitness patterns in general.

Despite the complexity of plant–herbivore interactions which might appear intractable to describe at first sight, we showed that simple patterns arise from two fundamental stochastic processes (equations (2.1) and (2.2)). All distributions of herbivore damage in nature belong to the same family, differing by only one parameter, the attack rate *λ*. This result provides a simple framework to understand patterns of damage, as well as the inequality of damage and its effects. For instance, where mean damage or per unit attack rate is lower, the higher damage inequality should select for more plastic, rapidly inducible defence in plants because defence allocation is otherwise difficult to match the cost of herbivore damage. This fact may explain why smaller plants and plants in temperate regions might be more inducible in nature [34]. More broadly, we can leverage well-established mean-centric plant defence theories to make predictions about the whole distributional pattern of damage [37–39]. Theories based on skewness, kurtosis or variance can then be nicely translated as an extension to existing herbivory theories [40–42], using the neutral model as a bridge between the mean and higher moments.

The remaining pattern not explained by neutral processes has several important implications. That herbivore damage is more regular among leaves than expected has long been hypothesized as a consequence of induced responses, which prevents successive herbivore feedings [2]. The regular spread of this herbivore damage within plants across organs can strongly ameliorate the negative effect of herbivory on individual plant fitness and growth by allowing for greater physiological compensation [4]. Further, the higher than predicted among-plant damage variability may reflect underlying variance in plant quality or variance in herbivore distribution, which we expect *a priori* to be generally aggregated [43]. Theoretical studies have shown that these overdispersed feeding patterns among plants can reduce temporal fluctuations and extinction risk of plant–herbivore populations, and increase equilibrium plant population density [5]. Finally, that observed damage patterns at the among-leaf scale resemble neutral patterns more closely than observed patterns at the among-plant scale could be explained by our neutral assumptions being more violated at the among-plant scale. This raises the intriguing possibility that to herbivores, different leaves within a plant may more be functionally equivalent despite some hypotheses of sub-individual trait variation as a plant defence [44,45] and optimal defence allocation [37].

A useful way forward to understanding plant–herbivore dynamics is not to abandon trait-based approaches, but to consider traits that affect the importance and nature of process stochasticity (table 2). Such understanding can inform the limits to the predictability of interactions and identify when conclusions drawn from simple models of plant–herbivore interactions apply. Such traits may include plant size, leaf lifespan, palatability, and apparency, that reduce the importance of stochasticity in herbivore damage by averaging over a greater number of herbivore attacks [26]. Further, if stochasticity itself can affect the fitness of plants and herbivores [2–5], then there can be selection on traits that modify the propensity for process stochasticity. For instance, larger plants sustain a greater number of attacks from herbivores, which should reduce the variability in damage (equation (2.3)). This means that plants that cannot cope with damage variability, such as those with a steep concave performance function along herbivore damage intensity, may have an advantage of being larger. Finally, understanding which traits can alter the process of plant–herbivore interactions beyond neutral expectations allows for greater prediction of important emergent properties of ensemble plant–herbivore interactions missed by traditional reductionist trait-based approaches (figure 2). Our finding that traits and phylogeny can predict differences between empirical and neutral patterns sheds optimistic light on this prospect.

Our study represents an essential step in critically evaluating observed herbivore damage patterns, identifying the contexts and manners by which observed damage patterns are different from neutral expectations, worthy of further investigation. Where our neutral model succeeds, it provides a useful approximation of natural damage patterns, and elsewhere, it may serve as a null against which future models that include heterogeneity in plant traits or herbivore behaviour may be compared. Our study highlights the importance of process-based stochasticity in shaping plant–herbivore interactions; the implications for plant defence evolution and herbivore population dynamics are yet almost entirely unexplored, despite their dominance in observed damage patterns. Just as different forms of process-based stochasticity have been incorporated into other fields, such as community ecology [31] and population genetics [46], plant–herbivore biologists too must embrace it as one of the core high-level processes that complements existing research programmes on plant–herbivore evolutionary and functional ecology.

## 4. Methods

### (a) Model parametrization

We fitted the neutral model to each dataset using maximum likelihood, estimating the unknown attack rate parameter *λ*. For among-leaf damage, we set the lower constraint *ϕ_m_* and upper constraint *ϕ_M_ a priori* as the lowest non-zero proportion damage value in our empirical dataset (0.5%) and the highest possible proportion damage (100%). We explore different values of the damage bounds and their caveats in electronic supplementary material, appendix, sensitivity analysis. For among-plant damage on a plant with *L* leaves, the bounds are multiplied by 1/*L*, as the area of a single leaf is a fraction of the whole plant leaf area. We used the median number of leaves sampled on each plant in a population as *L*.

### (b) Statistical analysis

Because our neutral model does not have a closed-form solution, we used Monte Carlo simulations in all our analyses. For each analysis, 100 predicted neutral distributions generated using the estimated $\hat{\lambda }$ and the original sample size were compared against the observed proportion damage and the results averaged. All analyses and simulations were performed in R (v. 4.2.1) [47]. Our code for the neutral model and an associated vignette are available in our custom R package *herbivar* (ver. 0.2.0) on GitHub.

To quantify how well the neutral model approximates observed data, for each dataset of proportion damage, we performed a KS test, which tests whether two samples are drawn from different distributions. We also compared ten summary statistics of the observed and predicted distribution collectively in redundancy analyses (RDA; package *vegan* [48]). These summary statistics, or probes, characterize key features of a distribution (mean, variance, skew, kurtosis, minimum, maximum, 25th quantile, 50th quantile, 75th quantile, Gini coefficient), providing informative diagnostics of model fit [29]. In the analysis at the among-leaf scale, survey ID was added as a conditional variable, thereby removing its effect in subsequent variance partitioning. Absolute model performance was assessed by examining the KS test statistic, the per cent of distributions with significant deviation from neutrality (as determined by the KS test), and the *r*^2^ of predicted versus observed CV of each dataset in a simple linear model. We log-transformed both predicted and observed CV to stabilize the variance and excluded surveys with zero variance. We also examined the constrained *R*^2^ of variation in standardized summary statistics from fitted RDA models. Lower proportion of variance constrained by whether a distribution is predicted or observed indicates better fit.

We quantified the relative fit of the neutral model against two plausible non-neutral, non-process-based null models, to allow for strong inference even if all models fit well by absolute measures. The zero-one-inflated beta distribution extends the beta distribution, commonly used to model proportion damage, to handle the presence of 0’s and 1’s in proportion damage data without the need for arbitrary transformations [49]. The hurdle-truncated-lognormal distribution has the density function

4.1 $$P(x;p_{0},\mu ,\sigma )=\{\begin{array}{c} \;p_{0},\;\\ (1-p_{0})(1-G(x=1;\mu ,\sigma )),\;\\ (1-p_{0})g(x;\mu ,\sigma ),\; \end{array}\;\begin{array}{c} x=0\;\\ x=1\;\\ x\neq 0,\;1.\; \end{array}$$

It extends the lognormal distribution with density function *g*(*x*;*μ*, *σ*) and cumulative distribution function *G*(*x*;*μ*, *σ*) to have probability mass at zero and one. The lognormal distribution is a reasonable candidate distribution as it can have a high positive skew and is a limiting distribution for repeated multiplicative processes. Both distributions were fitted to each dataset using maximum-likelihood estimation. We compared relative fits using sample size corrected Akaike information criterion (AICc).

To determine what empirical patterns the neutral model fails to predict, we examined the loading scores of the constrained axis of our RDA models. We also performed major axis regression analysis on log-transformed observed versus predicted CVs (package *lmodel2* [50]) to examine their relationship. Accurate prediction of observed CVs should have an intercept close to zero and a slope close to one. We did not consider regression coefficients from the ordinary least-squares regression models from which we obtained the *r*^2^ values because they downwardly bias the slope. We also compared the log-transformed CVs of observed, predicted, and shuffled distributions in two linear mixed models for among-leaf and among-plant damage (package *glmmTMB* [51]). We included the type of distribution (observed, predicted and shuffled) as a fixed effect and survey ID or plant ID nested within survey ID as random effects. The significance of marginal effects was tested with Wald *Z*-tests.

To identify the sources of deviations of neutral patterns from observation, we calculated the average KL divergence of predicted distributions from observed distributions across 100 bootstraps (package *philentropy* [52]). We found the species average KL divergence and measured the phylogenetic signal in Pagel's *λ*. A likelihood ratio test was used to assess the significance of the phylogenetic signal (package *phytools* [53]). We also fitted the KL divergence in a phylogenetic generalized linear mixed model, using a lognormal distribution and moderately regularizing priors (package *brms* [54]). We included absolute value of latitude, log plant height, mean annual temperature and mean annual precipitation as fixed effects. These predictors were *Z*-score transformed prior to fitting. Climate data were retrieved from WorldClim (ver. 2.1) [55] using survey site geocoordinates at a resolution of 2.5 min. Species identity and principal investigator (PI) identity, which account for repeated surveys of the same species and multiple surveys conducted by the same researcher, respectively, were included as random effects. For the analysis of KL divergence at the among-leaf scale, we added an additional random effect of survey identity nested within PI to account of the non-independence of individual plants within a survey. Phylogenetic relatedness was accounted for with a correlation structure assuming Brownian motion trait evolution. The phylogenetic tree was constructed by pruning from the most recently compiled mega-phylogenetic tree for vascular plants (package *V.PhyloMaker2*, Scenario 1 [56]). Variance partitioning of the sources of variation in KL divergences was done by calculating the change in residual-based *R*^2^ of selectively dropped regression terms.

## Data Availability

The data used in this manuscript are available on Dryad (https://doi.org/10.5061/dryad.qjq2bvqnz) [57]. The code used for the simulations and analyses is available on Zenodo (https://doi.org/10.5281/zenodo.10232775) [58]. The custom R package *herbivar* and a vignette on how to fit the neutral model to herbivory data are available via GitHub (https://github.com/vsbpan/herbivar). The supplemental analyses, methods and discussion are provided in electronic supplementary material [59].
